# Design and development of the DE-SPECT system: a clinical SPECT system for broadband multi-isotope imaging of peripheral vascular disease

**DOI:** 10.1088/1361-6560/ad5266

**Published:** 2024-06-11

**Authors:** E M Zannoni, P Sankar, Y Jin, C Liu, A J Sinusas, S D Metzler, L J Meng

**Affiliations:** 1 Department of Nuclear, Plasma, and Radiological Engineering, University of Illinois at Urbana Champaign, Urbana, IL, United States of America; 2 Department of Radiology, University of Pennsylvania, Philadelphia, PA, United States of America; 3 Department of Radiology and Biomedical Imaging, Yale University School of Medicine, New Haven, CT, United States of America; 4 Department of Medicine, Yale University School of Medicine, New Haven, CT, United States of America; 5 Beckman Institute for Advance Science and Technology, University of Illinois at Urbana-Champaign, Urbana, IL, United States of America

**Keywords:** organ-dedicated imaging, SPECT, CZT detectors, peripheral vascular disease

## Abstract

*Objective*. Peripheral Vascular Disease (PVD) affects more than 230 million people worldwide and is one of the leading causes of disability among people over age 60. Nowadays, PVD remains largely underdiagnosed and undertreated, and requires the development of tailored diagnostic approaches. We present the full design of the Dynamic Extremity SPECT (DE-SPECT) system, the first organ-dedicated SPECT system for lower extremity imaging, based on 1 cm thick Cadmium Zinc Telluride (CZT) spectrometers and a dynamic dual field-of-view (FOV) synthetic compound-eye (SCE) collimator. *Approach*. The proposed DE-SPECT detection system consists of 48 1 cm thick 3D-position-sensitive CZT spectrometers arranged in a partial ring of 59 cm in diameter in a checkerboard pattern. The detection system is coupled with a compact dynamic SCE collimator that allows the user to select between two different FOVs at any time during an imaging study: a wide-FOV (28 cm diameter) configuration for dual-leg or scout imaging or a high-resolution and high-sensitivity (HR-HS) FOV (16 cm diameter) for single-leg or focused imaging. *Main results.* The preliminary experimental data show that the CZT spectrometer achieves a 3D intrinsic spatial resolution of <0.75 mm FWHM and an excellent energy resolution over a broad energy range (2.6 keV FWHM at 218, 3.3 keV at 440 keV). From simulations, the wide-FOV configuration offers a 0.034% averaged sensitivity at 140 keV and <8 mm spatial resolution, whereas the HR-HS configuration presents a peak central sensitivity of 0.07% at 140 keV and a ∼5 mm spatial resolution. The dynamic SCE collimator enables the capability to perform joint reconstructions that would ensure an overall improvement in imaging performance. *Significance*. The DE-SPECT system is a stationary and high-performance SPECT system that offers an excellent spectroscopic performance with a unique computer-controlled dual-FOV imaging capability, and a relatively high sensitivity for multi-tracer and multi-functional SPECT imaging of the extremities.

## 1. Introduction

Peripheral Vascular Disease (PVD) is a slow and progressive circulatory disorder that results in vascular injury, inflammation, atherosclerosis, obstructive vascular disease, and microvascular dysfunction involving peripheral arteries and/or veins (Stacy and Sinusas 2016). PVD is most prevalent in the lower extremities, including peripheral arterial disease, chronic venous disease, chronic venous insufficiency, and deep vein thrombosis. PVD is a major cause of morbidity and mortality globally, affecting approximately 21 million Americans and an estimated 230 million people worldwide, with >50% of PVD patients being asymptomatic (Shu and Santulli 2018, Chou and Stacy 2020, Stacy 2022). PVD remains underdiagnosed and undertreated (Shu and Santulli 2018), resulting in life-altering claudication, non-healing ulcers, critical limb ischemia, limb amputation, and represents one of the leading causes of disability among people over age 60.

Current imaging techniques for PVD (Pollak *et al* 2012), such as computed tomography angiography and magnetic resonance angiography, are able to assess only gross anatomical changes or physiological status at the macrovascular level but fail to provide a complete evaluation at the molecular and microvascular level, needed for planning and optimizing PVD therapy (Stacy and Sinusas 2016). As emphasized by a recent careful review (Shabani Varaki *et al* 2018) of the existing diagnostic methods, the development of new non-invasive and efficient diagnostic approaches is crucial.

Multi-tracer Single Photon Emission Computed Tomography (SPECT) imaging is a molecular imaging technique that shows great potential in offering novel insights into the underlying pathophysiology of PVD (Stacy and Sinusas 2016, Stacy 2022). The approach involves the injection of multiple radiotracers with distinct gamma-ray energy photopeaks to image simultaneously different physiological processes in non-overlapping spectral energy windows. Considering the complex and multiple factors that contribute to the progression of PVD, the ability to assess quantitatively and simultaneously various molecular processes at the microvascular level could provide useful information in evaluating high-risk PVD patients, non-invasively monitoring their response to medical or surgical treatment, and potentially guiding therapeutic interventions (Stacy 2022).

Whereas several SPECT radiotracers are readily available in clinics for PVD applications (Stacy *et al* 2013, Chou and Stacy 2020), the success of multi-tracer SPECT imaging has been limited due to the relatively poor energy resolution of current clinical SPECT instrumentation based on traditional scintillator detectors (Sharir *et al* 2010, Karimeddini and Bergmann 2016). No organ-dedicated or tailored imaging systems for nuclear medicine applications is currently available to image lower extremities. By comparison, several CT and MRI systems for extremity imaging are specifically designed for hands, feet, ankles, and knees, and they are routinely used in clinical practice (Chung *et al* 2011, Carrino *et al* 2014, Sutter *et al* 2014).

In this paper, we report the design and Monte-Carlo-based performance evaluation of the Dynamic Extremity SPECT (DE-SPECT) system, the first SPECT system optimized for lower extremity imaging. The proposed DE-SPECT system is a stationary and high-performance scanner based on state-of-art 1 cm thick 3D position-sensitive cadmium zinc telluride (CZT) spectrometers with depth of interaction (DOI) capabilities and a dynamic synthetic compound-eye (SCE) collimator design (Lai and Meng 2018, Zannoni *et al* 2021). The detection system will offer an intrinsic resolution <0.75 mm along *X-, Y*-, and *Z*- directions and an excellent energy resolution over the 50–600 keV energy range, while a tailored C-shaped system geometry equipped with dynamic dual-field-of-view (FOV) collimators are designed to provide adequate sensitivity and imaging performance to perform dynamic and multi-functional SPECT imaging of PVD.

The purpose of this study is to present the DE-SPECT detection system and geometry, to report the design and prototype of the dynamic dual-FOV collimator, and to evaluate the expected system imaging performance through simulation studies.

## 2. Material and methods

### 2.1. 3D Position-sensitive CZT spectrometers

**Figure 1.**
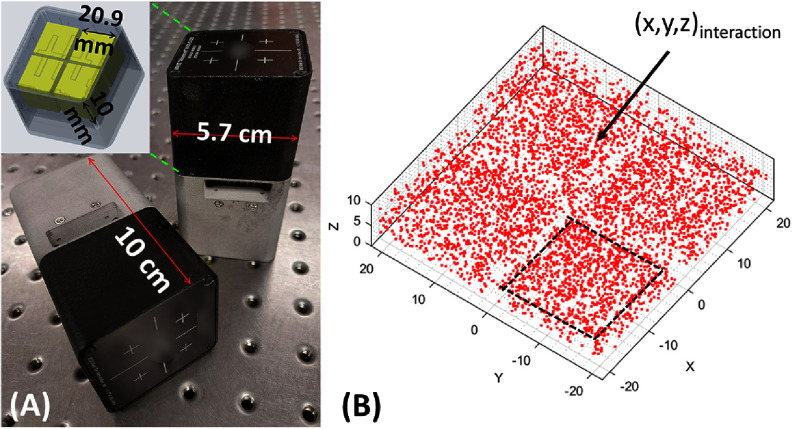
(A) A compact 3D position-sensitive CZT spectrometer. Zoomed view of the 2 × 2 CZT crystals. Adapted with permission from Zannoni *et al* (2022). (B) The interaction scatter plot shows 5000 interaction events acquired under flat illumination, proving the detector’s DOI capabilities. The four crystals are recognizable. Each red dot represents a gamma-ray interaction. Adapted with permission from Zannoni *et al* (2023).

The detection system of the DE-SPECT system consists of 48 3D-position sensitive CZT spectrometers, the M400i imager by H3D Inc., Ann Arbor, MI (figure 1(A)) (He *et al* 1999, Zhang *et al* 2012). Each detector module offers an effective area of ∼4.2 cm × 4.2 cm divided into four crystals of 20.9 × 20.9 × 10 mm^3^ in size (figure 1(A)), with 11 × 11 pixelated anodes of 1.9 mm pitch and a large planar cathode. Each crystal has a 0.55 mm wide guard ring, and a physical gap of 0.5 mm between crystals. A compact stainless-steel housing hosts the CZT crystals, the readout electronics, and the cooling system that allows performing acquisitions at room temperature.

For each gamma-ray interaction, the spectrometer provides 5D information *(x,y,z,t,E),* where *x* and *y* are the planar sub-pixel coordinates from the 11 × 11 pixelated anodes, *z* is the DOI in the 1 cm thick crystal, *t* is the interaction timestamp and *E* the gamma-ray energy. The (*x,y,z*) coordinates of each interaction event can be plotted in the 3D detector space as shown in the interaction scatter plot in figure 1(B). This shows 5000 counts acquired by the detector under flat illumination condition using a 70 *μ*Ci Co-57 point source.

To achieve sub-pixel resolution, the spectrometer readout electronics utilize the primary signal from the charge-collecting pixel and the transient charge induced on the surrounding pixels due to the weighting-potential crosstalk among anode pixels. The primary and transient signals are then used to locate each interaction with a spatial resolution smaller than the size of the anode pixels (Zhu *et al* 2011), while the interaction depth *z* is obtained from the ratio of the signals coming from the cathode and the anode (He *et al* 1999). This scheme has been shown to achieve a sub-pixel resolution of <0.5 mm FWHM in all three dimensions within a CZT crystal of up to 1.5 cm in thickness (Zhu *et al* 2011), showing an excellent and uniform energy resolution <1% FWHM at 662 keV for all four crystals in the detection unit (Zhang *et al* 2009). Further details on the detection unit can be found in (He *et al* 1999, Zhang *et al* 2009, 2012, Zhu *et al* 2011).

**Figure 2.**
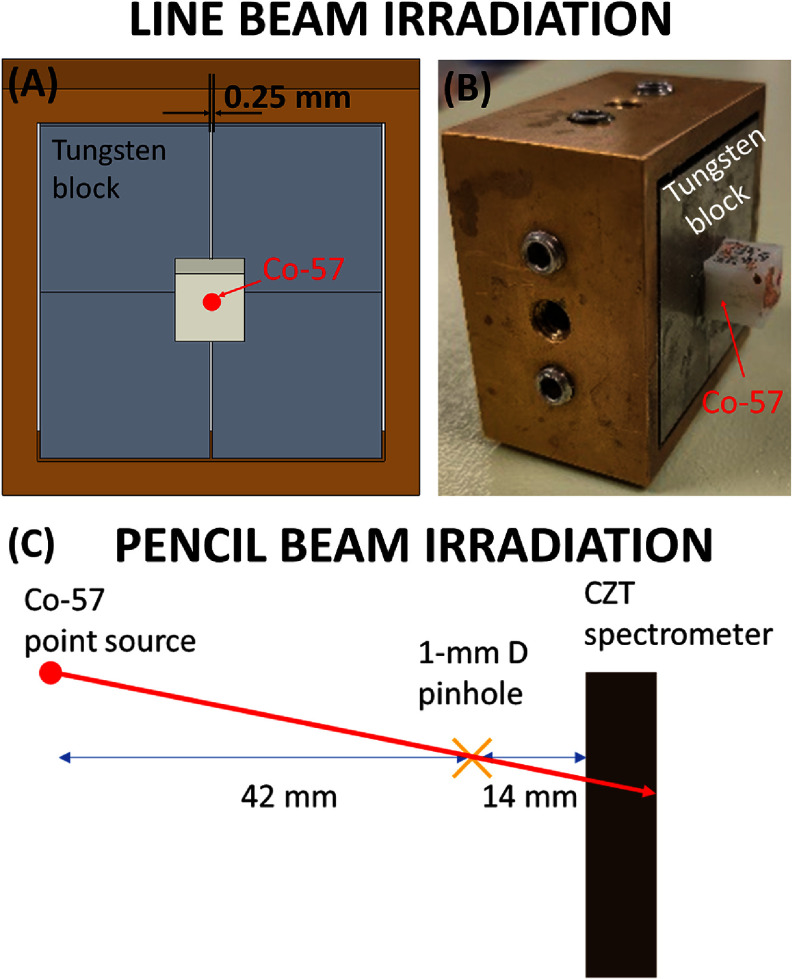
*Line beam irradiation:* (A) drawing and (B) setup of the 250 *μ*m-slit collimator and collimated Co-57 point source. (C) *Pencil beam irradiation:* diagram of the oblique incident 122 keV pencil beam.

As preliminary tests of the detector performance, we quantitively characterized the spatial resolution of a CZT spectrometer along the *X-, Y*-, and *Z*- directions. We performed the following tests using a Co-57 point source of 70 *μ*Ci with a radioactive core of 0.25 mm in diameter: (a) *in a line beam irradiation setup* (figures 2(A) and (B)), we collimated the point source using a parallel-hole slit tungsten collimator of 0.25 mm width, 40 mm length and 30 mm thickness (figures 2(A) and (B)) to produce a 0.25 mm line beam. The collimator used (figure 2(A)) presents four straight blocks of tungsten and a system of side screws that allow to precisely define the width of the slit. The distance from the slit collimator to the detector surface was 7 mm, including 5 mm of internal gap between the detector mask and the crystal surface and 2 mm of mask thickness. (b) *In a pencil beam irradiation setup* (figure 2(C)), we collimated the point source using a 1 mm diameter pinhole, and we irradiated the detector with an oblique incident pencil beam, according to the geometry shown in figure 2(C).

To demonstrate the spectroscopic performance of the CZT detectors, we acquired 4 h from an uncollimated liquid source of 0.1 mCi Ac-225 in 0.2 mL in a glass vial using a single CZT spectrometer in flat illumination conditions. In the context of targeted alpha-emitter therapy (Seo 2019, Nelson *et al* 2020), Ac-225 represents a promising radioisotope as a radiotherapeutic agent for neuroendocrine tumors, especially for metastatic prostate cancer (Scheinberg and McDevitt 2011, Kruijff *et al* 2019, Ocak *et al* 2020). Ac-225 is not used in PVD applications, but it was chosen due to its complex decay scheme and densely populated energy spectrum that requires an excellent energy resolution from the detection system (Scheinberg and McDevitt 2011). Ac-225 presents a long decay chain including 4 *α*- and 3 *β*-emitting disintegrations, two useful gamma emissions for imaging (218 keV from Fr-221 and 440 keV from Bi-213), and many characteristic x-rays (86.1 keV from Fr-221, 100 keV from Ac-225, 117.3 keV from Tl-209, etc).

### 2.2. Organ-dedicated system design

To the best of our knowledge, the DE-SPECT is the first organ-dedicated SPECT system being developed for imaging lower extremities. Whereas many organ-dedicated SPECT systems have been developed for brain, breast, and cardiac imaging applications (González *et al* 2018), molecular imaging of the extremities still relies on large FOV scanners with non-optimized geometries. Organ-dedicated molecular imaging scanners offer some desirable features such as higher sensitivity and angular coverage by placing the detectors closer to the organ in an optimized geometry, improved spatial resolution, better image contrast recovery by reducing the noise from other organs, smaller footprints, and overall lower cost (González *et al* 2018).

**Figure 3.**
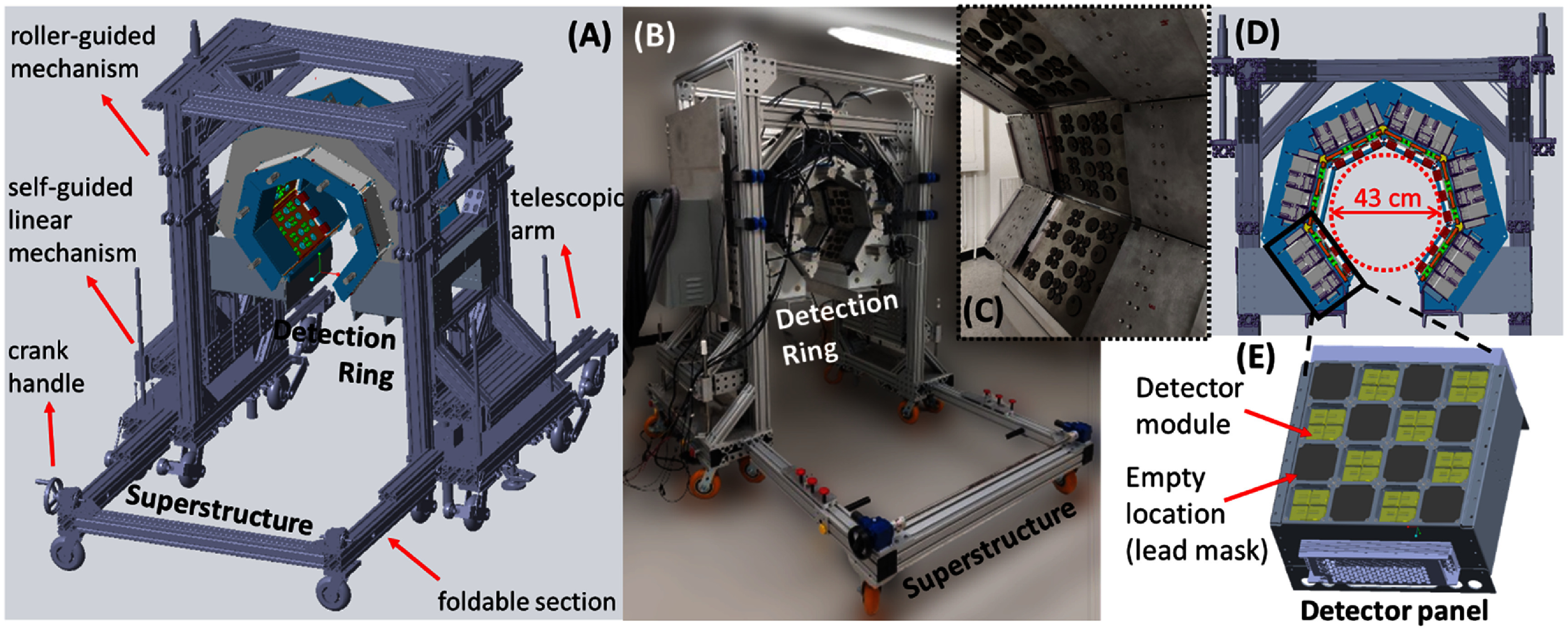
The DE-SPECT system: (A) 3D rendering of the DE-SPECT system. (B) The assembled DE-SPECT system. (C) Gantry details. (D) Trans-axial section of the detection ring with internal details. (E) A single detector panel (4 × 4 array) half-populated in a checkerboard pattern (detector crystals in yellow), the empty positions are shielded with 1.27 cm thick lead masks.

The DE-SPECT system design (figures 3(A)–(D)) consists of six stationary detector panels arranged in a C-shaped partial ring of ∼59 cm in diameter with no collimator and ∼43 cm in diameter with collimator installed (figure 3(D)). A bottom opening of 30 cm allows the positioning of the patient’s leg(s). Each panel can accommodate up to 16 CZT spectrometers arranged in a 4 × 4 array. In the current design, each detector panel is half-populated with 8 detector modules in a checkerboard pattern (figure 3(E)), offering an overall active area of 838.7 cm^2^. The empty detector positions are masked with 1.27 cm thick lead pieces for shielding.

The detection ring is installed on an adjustable and portable superstructure (figures 3(A) and (B)), produced using off-the-shelf 80/20 extruded aluminum profiles and accessories. The wheeled superstructure, designed to fit inside hospital elevators and through doorways, will allow to easily move the DE-SPECT in and out of the imaging suite, and drive the system to the patient’s bed of a pre-existing cardiac SPECT(/CT) scanner to perform simultaneous imaging of the lower extremities and the heart. The superstructure allows the vertical adjustment of ±100 mm of the gantry position that will be elevated above the patient’s bed of the cardiac scanner with minimal clearance. In this way, an elevated leg of a supine patient will be positioned in the DE-SPECT imaging FOV using customized leg elevation pillows, providing sufficient comfort to the patient for the duration of the scan.

Special design choices were made to facilitate the portability and integration of the DE-SPECT system with pre-existing instrumentation. Four self-locking worm-gear screw jacks are coupled to a crank handle, allowing lifting of the system with a total travel range of 200 mm. Two self-guided linear mechanisms on each side of the scanner ensure that the travel plane is perpendicular to the bed surface. Further, a pair of roller-guided mechanisms in the front and back of the scanner reduces tip and tilt hazards that might occur during the scanner transportation or during the vertical height alignments.

A total of 12 swivel caster wheels in the superstructure allow easy maneuvering for transportation and alignment. On the back side, a telescopic system is used to provide stability and minimize the gap between the DE-SPECT system and the pre-existing SPECT(/CT) scanner. On the front, the superstructure presents a foldable system to reduce the axial length of the gantry to 90 cm to clear doors and elevators.

### 2.3. Dynamic dual-field-of-view synthetic compound-eye collimators

**Figure 4.**
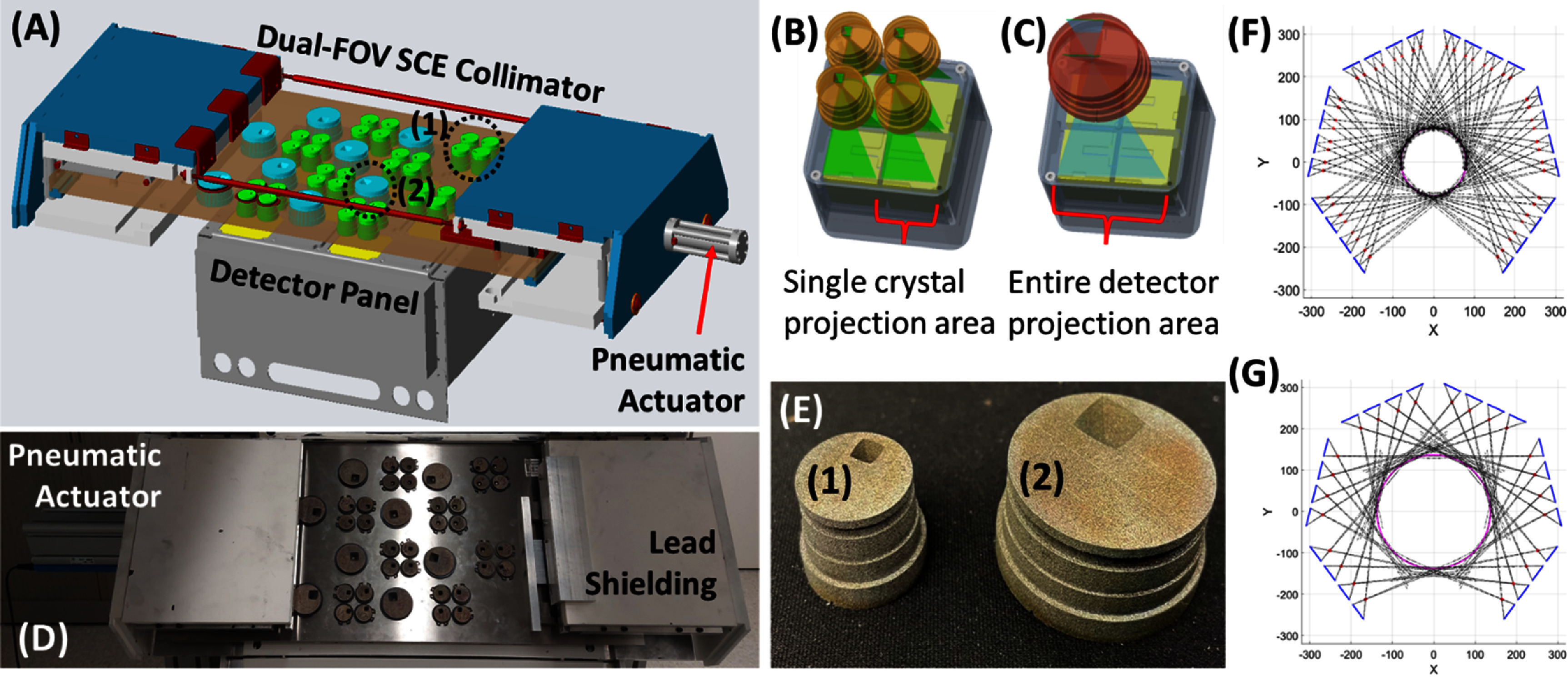
The dynamic dual-FOV synthetic compound-eye collimator: (A) A dynamic SCE collimator module coupled with a detector panel. Two sets of apertures are available, green (type 1) and cyan (type 2). (B) Design of a 2 mm D lofthole (type 1) projecting on a single crystal. Adapted with permission from Sankar *et al* (2022). (C) Design of a 3 mm D lofthole (type 2) projecting on 2 × 2 crystals in a detector module. Adapted with permission from Sankar *et al* (2022). (D) Prototype of the dual-FOV SCE panel. (E) 3D printed inserts produced with tungsten laser melting. (F) Line diagram of the DE-SPECT design in the HR-HS FOV imaging configuration. Adapted with permission from Sankar *et al* (2022). (G) Line diagram of the DE-SPECT design in the wide-FOV imaging configuration. (legend: blue solid line—detector surface, red star—lofthole center, black dashed line—lofthole FOV line). Adapted with permission from Sankar *et al* (2022).

Each CZT detector panel is coupled with a dynamic dual-FOV SCE collimator that consists of a dense 2D array of narrow-open-angle apertures (figures 4(A) and (D)). Each aperture projects an independent view of the FOV onto the surface of the corresponding high-resolution detector module (figures 4(B) and (C)) (Lai and Meng 2018, Zannoni *et al* 2021, Meng *et al* 2022). The design does not allow projection multiplexing.

The dynamic dual-FOV SCE collimator utilizes the checkerboard pattern in the detector panels to allow two sets of collimators to be alternatively positioned in front of the CZT detector modules (green and cyan inserts in figure 4(A), and corresponding 3D printed inserts in figure 4(E)). The first set consists of 192 loftholes, each 2 mm in diameter, focusing on an HR-HS FOV of 16 cm in diameter for single-leg imaging. In this configuration, each insert focuses on a single 20.9 × 20.9 mm^2^ crystal of the detector module (figures 4(B) and (F)). The second set offers 48 loftholes, each 3 mm in diameter, focusing on a wide FOV (28 cm diameter) for dual-leg imaging. In this configuration, each insert focuses on the entire 2 × 2 crystals inside a single detector module (figures 4(C) and (G)). The lofthole inserts are produced using rapid additive manufacturing with selective laser melting of tungsten powder (figure 4(E)) and are mounted on a 1.27 cm thick tungsten plate (figure 4(D)).

In both designs, a minification factor 1:8 is used. The combination of the highly de-magnifying geometry and the large number of independent micro-camera-elements is one of the main features of an SCE collimator (Zannoni *et al* 2021), with the objectives to reduce the total CZT detector volume needed in comparison to magnifying geometries and to boost the angular sampling with densely packed independent cameras. To achieve this, pixelated detectors with sub-mm resolution are required.

In the DE-SPECT design, the lofthole aperture (Deprez *et al* 2013) has been preferred over a traditional pinhole for optimal usage of the detector’s active area. Loftholes indeed present square exiting profiles that efficiently tile the projections onto the squared detector surface, resulting in a higher detection efficiency in comparison to the circular-shaped profiles of pinholes.

The DE-SPECT C-shaped detection system offers an angular coverage of 303° around the gantry axis, with a total of 48 (in wide-FOV mode) or 192 (in HR-HS mode) independent camera elements that assure an extensive angular sampling for dynamic imaging of the lower extremities in patients with PVD. During an imaging acquisition, the wide-FOV mode will be used in case of dual-leg imaging or to acquire a scout image and to identify the region of interest (ROI), and then the user can switch to HR-HS mode for single-leg acquisition or focused imaging in the ROI. To support the patient’s leg(s) during the imaging session, an inflatable leg elevation pillow or mattress will be used. We will also consider designing a supporting structure with brackets for each leg that will allow us to independently position each leg in the FOVs of the DE-SPECT system.

In each detector panel, a computer-controlled position-sensitive pneumatic linear actuator (figures 4(A) and (D)) slides the tungsten plate axially to interchange on the fly (<5 s) the aperture set. This precise and fast-switching mechanism places the desired apertures in front of the CZT detector modules in the checkerboard pattern. Mechanical stops guarantee reproducibility when the tungsten plates translate 57 mm axially between the two imaging configurations.

### 2.4. System performance evaluation

#### 2.4.1. System response function and image reconstruction

To evaluate the system performance of the proposed DE-SPECT design through simulations, the system response functions (SRF) in the wide-FOV and in the HR-HS configuration were separately estimated. The discretized version of the SRF, referred to as the system response matrix (SRM) (Frey and Tsui 2006), is defined as

\begin{align*}{\boldsymbol{A}} = \left[ {\begin{array}{*{20}{c}} {{a_{11}}}&amp; \cdots &amp;{{a_{1N}}} \\ \vdots &amp; \ddots &amp; \vdots \\ {{a_{M1}}}&amp; \cdots &amp;{{a_{MN}}} \end{array}} \right]\end{align*}

where each element ${a_{mn}}$ gives the probability of a gamma-ray emitted at the *n*th source voxel in the object space and being detected by the *m*th detector pixel within a unit of imaging time. To estimate the pixel intensities in projection data ${\boldsymbol{y}} = \left[ {\begin{array}{*{20}{c}} {{y_1}}&amp; \ldots &amp;{{M_m}} \end{array}} \right],$ with *M* the total number of detector pixels, the following operation is performed:

\begin{align*}E\left[ {\,\boldsymbol{y}} \right] = {\boldsymbol{Ax}}\end{align*}

where ${\boldsymbol{x}} = \left[ {\begin{array}{*{20}{c}} {{x_1}}&amp; \ldots &amp;{{x_N}} \end{array}} \right]{\text{ }}$ denotes the unknown values of the voxels in the object space, with *N* the total number of voxels.

To calculate the SRM, we used a voxel-driven method (Frey and Tsui 2006) that we have developed in C language and previously implemented (Lai and Meng 2018, Lai *et al* 2020, Zannoni *et al* 2021). Gamma rays are traced from the center of each voxel in the object space to the center of each detector pixel through the reconstruction volume, the collimator and the CZT volume itself.

The object space is discretized in 180 × 180 × 180 cubic voxels of 2 × 2 × 2 mm^3^. The simulated CZT detectors present the same properties as the physical spectrometer described in 2.1. Each detector has 4 crystals with inter-crystal gaps of 1.6 mm along the vertical and horizontal directions. Each crystal is divided in a 3D matrix of 28 (X) × 28 (Y) × 10 (Z) voxels of 750 *μ*m × 750 *μ*m × 1 mm in size, assuming a realistic DOI resolution of 1 mm. The positions of the single detector modules and of the detector panels are known from the design geometry, and they are kept the same in the wide-FOV and the HR-HS configuration. The difference between the two SRMs resides in the two collimators simulated: in the wide-FOV configuration 48 tungsten loftholes, each 3 mm in diameter (figure 4(E), right), are coupled with an entire CZT detector module, in the HR-HS configuration 192 tungsten loftholes, each 2 mm in diameter (figure 4(E), left), are coupled with each CZT crystal according to the geometry shown in figures 4(B) and (C).

The estimated SRMs include the attenuation of gamma rays through the collimator, especially in the knife-edge region, and through the CZT volume including the detector response at 140 keV (97% probability of interaction for 1 cm thick CZT crystal), but it does not model scattered gamma rays and the attenuation in the object. The scatter component is not modeled under the assumption that the superior energy resolution of the spectrometers allows us to choose narrower energy windows centered in the photopeaks, and therefore reduce the contribution from scattered gamma-rays in the reconstructed images. This will be handled as an additive term in the expected value and estimated using energy-based methods (Popescu *et al* 2006, Efthimiou *et al* 2022).

Once the SRMs are computed and stored, 48 mean projections are calculated by forward-projection according to (2) in the case of wide-FOV mode, 192 in case of HR-HS mode. The noisy projections are then obtained by adding Poisson noise to the mean values using the *poidev* function in C (Press *et al* 2007). For image reconstruction, we implemented the 3D ordered subset expectation maximization (OSEM) algorithm (Hudson and Larkin 1994) with eight subsets. In this study, no filter or regularization are applied.

#### 2.4.2. Joint image reconstruction

The flexibility introduced by the dynamic dual-FOV SCE collimator allows the user to carry out joint image reconstructions using the projections acquired with both the wide-FOV and in the HR-HS configuration. Joint image reconstruction methods have been recently explored for Compton imaging (Roser *et al* 2022) and in several SPECT imaging applications, such as ictal/interictal SPECT imaging (Rakvongthai *et al* 2017), rest/stress myocardial perfusion SPECT (Lai *et al* 2018) and Y-90 Bremsstrahlung SPECT (Chun *et al* 2020), consistently showing an improvement in the imaging performance and quantitation.

In a generic case where an imaging system can assume *L* imaging configurations, the general expression of the forward model in matrix-vector format can be formulated as follows:

\begin{align*}\left( {\begin{array}{*{20}{c}} {\begin{array}{*{20}{c}} {\overline {{{\boldsymbol{y}}_{\boldsymbol{1}}}} } \\ {\overline {{{\boldsymbol{y}}_{\boldsymbol{2}}}} } \end{array}} \\ \vdots \\ {\overline {{{\boldsymbol{y}}_{\boldsymbol{L}}}} } \end{array}} \right) = \left( {\begin{array}{*{20}{c}} {\begin{array}{*{20}{c}} {{w_1}{{\boldsymbol{A}}_{\boldsymbol{1}}}}&amp;{\boldsymbol{0}} \\ {\boldsymbol{0}}&amp;{{w_2}{{\boldsymbol{A}}_{\boldsymbol{2}}}} \end{array}{\text{ }}\begin{array}{*{20}{c}} {\boldsymbol{0}}&amp;{{\text{ }}{\boldsymbol{0}}} \\ \ldots &amp;{{\text{ }}{\boldsymbol{0}}} \end{array}} \\ {{\text{ }}\begin{array}{*{20}{c}} \vdots &amp; \vdots \\ {{\text{ }}{\boldsymbol{0}}}&amp;{{\text{ }}{\boldsymbol{0}}} \end{array}{\text{ }}\begin{array}{*{20}{c}} \ddots &amp; \vdots \\ \ldots &amp;{{w_{\boldsymbol{L}}}{{\boldsymbol{A}}_{\boldsymbol{L}}}} \end{array}} \end{array}} \right){\boldsymbol{x}}\end{align*}

where $\overline {{{\boldsymbol{y}}_{{\boldsymbol{1}} \ldots {\boldsymbol{L}}}}} $ (*M* × 1 vectors) are the estimated projections obtained from the imaging system in a specific configuration described by the corresponding SRM ${{\boldsymbol{A}}_{{\boldsymbol{1}} \ldots {\boldsymbol{L}}}}$. *x* (*N* × 1 vector) are the unknown values in the object space and ${w_{1 \ldots L}}$ are explicit parameters that control the weight of each imaging configuration in the reconstruction. This is equivalent to acquiring data with different scan durations in each imaging configuration.

In the current work, the DE-SPECT system allows for two exchangeable imaging configurations, therefore we adopted a basic joint method that concatenates the two SRM’s from (3). This is described by the simplified forward model:

\begin{align*}\left[ {\begin{array}{*{20}{c}} {{{{\bar {\boldsymbol{y}}}}_{{\text{WF}}}}} \\ {{{{\bar {\boldsymbol{y}}}}_{{\text{HR}} - {\text{HS}}}}} \end{array}} \right] = \left[ {\begin{array}{*{20}{c}} {{w_{{\text{WF}}}}{\boldsymbol{A}_{{\text{WF}}}}} \\ {{w_{{\text{HR}} - {\text{HS}}}}{\boldsymbol{A}_{{\text{HR}} - {\text{HS}}}}} \end{array}} \right]\boldsymbol{x}\end{align*}

where ${{\bar {\boldsymbol{y}}}_{{\text{WF}}}}$ (*M ×* 1 vector) and ${{\bar {\boldsymbol{y}}}_{{\text{HR}} - {\text{HS}}}}$ (*M ×* 1 vector) are the estimated projections in the wide-FOV and HR-HS configuration, respectively. Similarly, ${\boldsymbol{A}_{{\text{WF}}}}$ (*M × N* matrix) and ${\boldsymbol{A}_{{\text{HR}} - {\text{HS}}}}$ (*M × N* matrix) are the SRM’s in the wide-FOV and HR-HS configuration, respectively, and the ${w_{{\text{WF}}}}$ and ${w_{{\text{HR}} - {\text{HS}}}}$ parameters define the time spent in each imaging configuration. For simplicity, we will consider that the total scanning time is equally split between the two configurations.

The OSEM algorithm from iteration *n* to *n* + 1 can be then rewritten as :

\begin{align*}{{\boldsymbol{x}}^{\left( {n + 1} \right)}} = \frac{{{{\boldsymbol{x}}^{\left( n \right)}}}}{{{{\left[ {\begin{array}{*{20}{c}} {{w_{{\text{WF}}}}{\boldsymbol{A}_{{\text{WF}}}}} \\ {{w_{{\text{HR}} - {\text{HS}}}}{\boldsymbol{A}_{{\text{HR}} - {\text{HS}}}}} \end{array}} \right]}^T}\left[ {\begin{array}{*{20}{c}} 1 \\ 1 \end{array}} \right]}}{\left[ {\begin{array}{*{20}{c}} {{w_{{\text{WF}}}}{\boldsymbol{A}_{{\text{WF}}}}} \\ {{w_{{\text{HR}} - {\text{HS}}}}{\text{ }}{\boldsymbol{A}_{{\text{HR}} - {\text{HS}}}}} \end{array}} \right]^T}\frac{{\left[ {\begin{array}{*{20}{c}} {{{{\bar {\boldsymbol{y}}}}_{{\text{WF}}}}} \\ {{{{\bar {\boldsymbol{y}}}}_{{\text{HR}} - {\text{HS}}}}} \end{array}} \right]}}{{\left[ {\begin{array}{*{20}{c}} {{w_{{\text{WF}}}}{\boldsymbol{A}_{{\text{WF}}}}} \\ {{w_{{\text{HR}} - {\text{HS}}}}{\text{ }}{\boldsymbol{A}_{{\text{HR}} - {\text{HS}}}}} \end{array}} \right]{x^{\left( n \right)}}}}.\end{align*}

#### 2.4.3. Phantom studies

To evaluate the performance of the DE-SPECT system design in the two collimator configurations, the following digital phantoms were used as detailed below.

(A) **Defrise phantoms.** Two Defrise phantoms were simulated under noiseless conditions to evaluate the axial sampling of the system in the two collimator configurations: a cylinder of 24 cm diameter and 18 cm length, divided in 6 mm thick disks and spacings was used in the wide-FOV configuration, whereas a cylinder of 16 cm diameter and 10 cm length divided in 4 mm thick disks and spacings was used in the HR-HS configuration.
(B) **Hot-rod phantoms.** Two hot-rod phantoms were used to assess the different spatial resolution in the two collimator configurations under noisy and noiseless conditions. For the wide-FOV configuration, the phantom has four groups of hot-rods with diameters ranging from 6 to 12 mm and 20 cm in length, placed inside a cylinder with a diameter of 26 cm and axial length of 20 cm. It contains 10 mCi of Tc-99 m in total with a signal-to-background ratio (S/B) of 10:1. For the HR-HS configuration, the diameters of the four hot-rod range from 4 to 10 mm, placed inside a cylinder with a 16 cm diameter and 10 cm axial length. Phantom contains a total of 2.5 mCi Tc-99 m with S/B 10:1. In both phantoms, the distance between the center of two neighboring rods is twice their diameter, and they are imaged for 1 h in case of reconstructions in a single mode reconstruction, 30 min in case of joint reconstruction.

For all phantoms, the main axis was placed along the gantry axis in the simulations.

#### 2.4.4. Quantitative performance metrics

We used standard quantitative metrics to evaluate the imaging performance for the two collimator configurations.

(A) **Normalized root-mean-square error (NRMSE).** NRMSE is used to estimate the quantitative accuracy between the reconstructed images and the phantom in the OSEM reconstruction. It is defined as
  \begin{align*}{\text{NRMSE}} = \sqrt {\frac{{\mathop \sum \nolimits _{n = 1}^N{{\left( {{x_n} - x_n^T} \right)}^2}}}{{\mathop \sum \nolimits _{n = 1}^Nx{{_n^T}^2}}}} {\text{ }}\end{align*}
  where ${x_n}$ is the reconstructed value for the *n*th voxel in the image, $x_n^T$ the true value of the *n*th voxel, and *N* is the total number of voxels. The NRMSE is calculated for each iteration in the OSEM reconstruction. In case of reconstruction under noisy conditions, we choose the image at the iteration where the NRMSE reaches the minimum. In case of reconstruction under noiseless conditions, the NRMSE decreases asymptotically, and we choose the image at the iteration where the difference in the NRMSE with the previous one is less than 0.1‰.
(B) **Contrast recovery coefficient (CRC).** The CRC (Van Audenhaege *et al* 2013) was calculated in the hot-rod phantoms for assessment of the contrast recovery. The CRC for one hot-rod of each group (the one closest to the phantom central axis) was estimated as
  \begin{align*}{\text{CRC}}{\text{ }}\left( \% \right) = {\raise0.7ex\hbox{${\left( {\frac{{{{\bar x}_{{\text{rodj}}}} - {{\bar x}_{{\text{bck}}}}}}{{{{\bar x}_{{\text{bck}}}}}}} \right)}$} \!\mathord{\left/ {\vphantom {{\left( {\frac{{{{\bar x}_{{\text{rodj}}}} - {{\bar x}_{{\text{bck}}}}}}{{{{\bar x}_{{\text{bck}}}}}}} \right)} {\left( {C - 1} \right)}}}\right.} \!\lower0.7ex\hbox{${\left( {C - 1} \right)}$}} \times 100.\end{align*}
  where ${\bar x_{{\text{rodj}}}}$ is the mean count in the hot rod *j*, ${\bar x_{{\text{bck}}}}$ is the mean count in the background rod (8 × 8 voxels rod in the background region of the phantom) and *C* the true rod–background ratio from the digital phantom (*C* = 10).
(C) **Contrast-to-noise ratio (CNR).** Similarly, in the hot-rod phantoms, the CNR was calculated as
  \begin{align*}{\text{CNR}} = {\text{ }}\frac{{\left| {{{\bar x}_{{\text{rodj}}}} - {{\bar x}_{{\text{bck}}}}} \right|}}{{{\sigma _{{\text{bck}}}}}}\end{align*}
  where ${\sigma _{{\text{bck}}}}$ is the standard deviation of the counts in the background rod.
(D) **Noise coefficient (NC)**. NC (Van Audenhaege *et al* 2013) was calculated as
  \begin{align*}{\text{NC }}\left( \% \right) = \frac{{{\sigma _{{\text{bck}}}}}}{{{{\bar x}_{{\text{bck}}}}}} \times 100\end{align*}
  and used to plot the CRC-NC curves to compare the two system configurations, in terms of image contrast recovery and noise amplification during the iteration reconstruction process.

## 3. Results

### 3.1. Preliminary detector performance

The preliminary experimental data acquired with a CZT spectrometer demonstrated a sub-0.75 mm intrinsic spatial resolution along *X-, Y*-, and *Z*- directions, and an excellent spectroscopic performance over a broad energy spectrum (50–600 keV). The detector unit outputs the (*x,y,z*) coordinates of each interaction point with a 0.01 mm stamp that is used to plot and process the following results.

**Figure 5.**
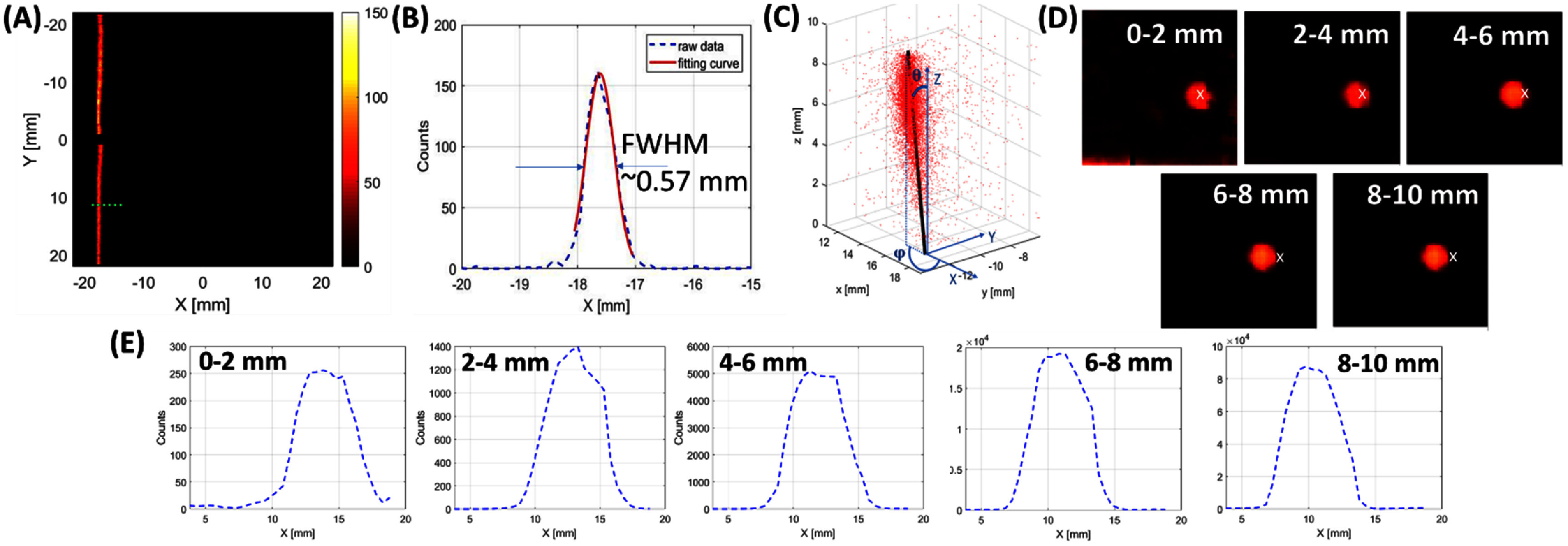
Line beam irradiation: (A) projection of the 122 keV line beam in the energy window [120, 125 keV] and with compressed DOI information, (B) 1D profile of the green dashed line in (A) present FWHM of 0.57 mm. Pencil beam irradiation: (C) scatter plot of the gamma-ray interactions (red dots) from the incident 122 keV pencil beam. The black line represents the incident beam direction which is fitted using the least-square method with depth weighting due to the detector material attenuation. (D) Accumulated projections (from anode to cathode) acquired in each 2 mm layer of the 1 cm CZT crystal. The white cross is the weighting center from the first projection. (E) The corresponding central profiles from figure 5(D).

From the Co-57 line beam irradiation, the projection on the CZT spectrometer is shown in figure 5(A) with compressed DOI information. In this case, only the (*x,y*) coordinates of the interaction points are used to produce the planar projection. The 1D profile (green dashed line in figure 5(A)) presents a FWHM of 0.57 mm estimated with Gaussian fitting (figure 5(B)). The small gap along the projection is due to the non-active gap (physical and guard ring) of 1.6 mm between crystals. Similar performance is seen along the *y*-direction. Since the extrinsic resolution *R*_ext_ is

\begin{equation*}{R_{{\text{ext}}}} = \sqrt {R_{{\text{int}}}^2 + R_{{\text{coll}}}^2} \end{equation*}

where *R*_int_ is the intrinsic detector resolution and *R*_coll_ is the collimator resolution equal to the slit width in this case, *R*_int_ results to be 0.512 mm. Based on these experimental results, a pixel size of 750 *μ*m × 750 *μ*m is conservatively chosen for the simulation studies, as reported in 2.4.1.

From the Co-57 oblique incident pencil beam irradiation, the data collected is visualized as scatter plot of the gamma-ray interactions in figure 5(C). *Z* = 10 mm is the detector front surface (cathode), therefore, the number of counts decreases with *Z* decreasing due to material attenuation. The black line in figure 5(C) represents the oblique incident beam having polar angle *θ* of 32.8° and azimuthal angle ϕ of 174.5°. The line is fitted using the least-square method in MATLAB with depth weighting to consider the detector material attenuation. Finally, figure 5(D) shows the accumulated projections from the Co-57 point source acquired in each 2 mm layer (from anode to cathode) of the 1 cm thick CZT crystal. The corresponding central profiles are shown in figure 5(E) and the FWHMs are (from anode to cathode): 4.61, 4.55, 4.50, 4.33, 4.27 mm, and the *R*_int_ are 4.50, 4.44, 4.38, 4.22 and 4.15 mm, respectively. The DOI capability of the spectrometer shows how the projections shift in the different layers, where the white cross represents the weighting center from the first projection. This feature would allow to correct for the parallax error in oblique incident beams, improving the reconstructed spatial resolution. It is worth nothing that given the divergence of the gamma-ray beams in the plane parallel to the cross-section of the slit and pinhole, the widths of the line and pencil beam reaching the surface of the detector and at different depths would be wider than 250 *μ*m and 1 mm, respectively. We are unable to determine the actual widths of the beams at the surface of the detector and at different depths due to the lack of the precise dimension of the commercial point source and the actual distribution of the activity within the active source volume. Therefore, the intrinsic resolution calculated with (10) is an underestimate of the actual intrinsic resolution of the detector.

**Figure 6.**
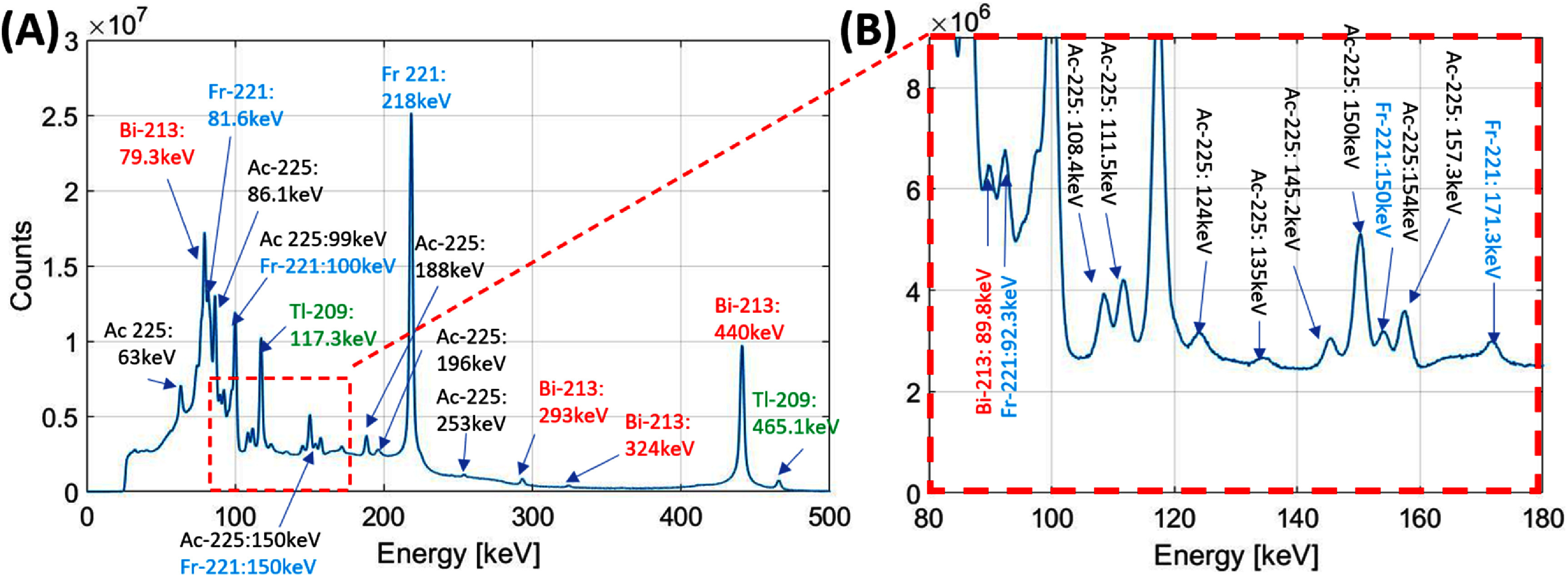
(A) Energy spectrum in the [50, 500] keV range acquired from a liquid source of 0.1 mCi Ac-225 in 0.2 mL in a glass vial using a single CZT spectrometer in flat illumination conditions. The energy peaks from different radioisotopes are highlighted with different colors. (B) Zoomed view in the [80, 180] keV range.

Finally, figure 6 shows the energy spectrum of Ac-225 acquired with a single CZT spectrometer. The excellent energy resolution allows to resolve and identify primary and secondary gamma-ray contributions from Ac-225 (100 and 150 keV) and its daughters, Fr-221 (218 keV), Bi-213 (79.3, 440 keV), Tl-209 (79.3, 117.3, 440 keV). Additional details are shown in figure 6(B). The energy resolutions from the main peaks are: 2.1 keV FWHM at 117.3 keV (Tl-209), 2.6 keV FWHM at 218 keV (Fr-221) and 3.3 keV FWHM at 440 keV (Bi-213).

### 3.2. System sensitivity and field-of-view

The system sensitivity in the two imaging configurations is estimated using the corresponding SRM in (1), where the sensitivity value of each voxel *i* in the object-space is

\begin{align*}{s_i} = \mathop \sum \limits_{j = 1}^M {a_{ij{\text{ }}}}.\end{align*}

**Figure 7.**
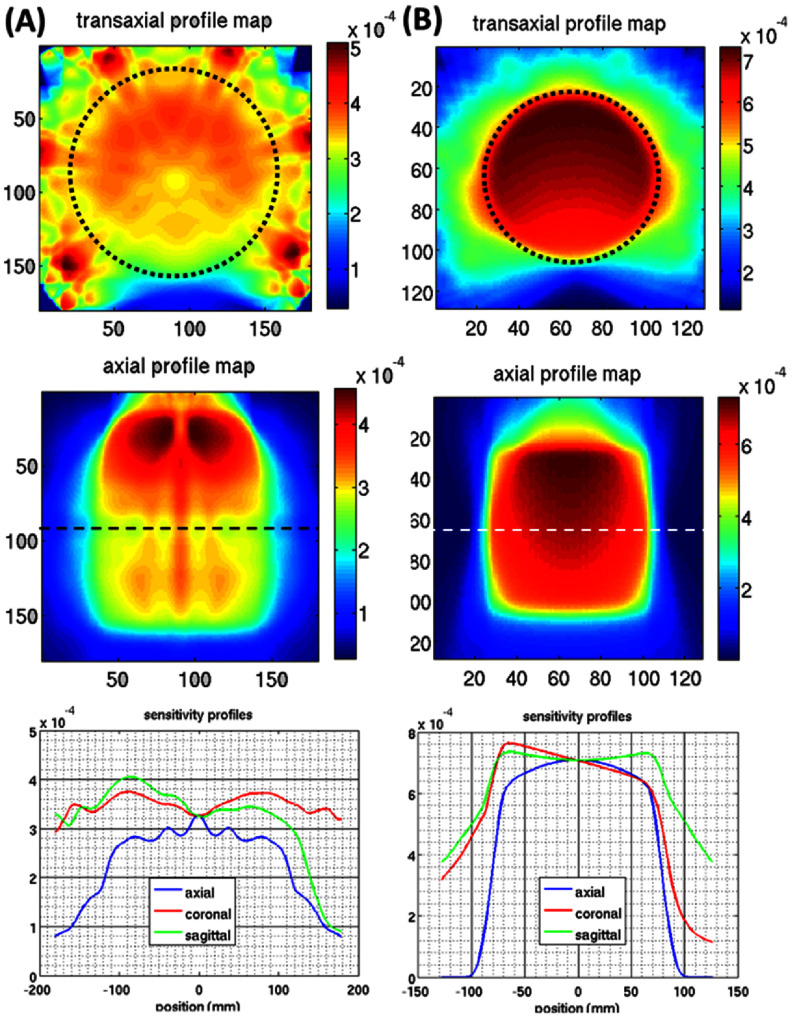
The DE-SPECT system sensitivity: (A) wide-FOV imaging configuration and (B) HR-HS FOV imaging configuration. (1st row) Trans-axial and (2nd row) axial sensitivity maps, (3rd row) sensitivity profiles. The circled dotted lines show the transaxial FOV, and the dashed lines show the gantry axis.

The sensitivity maps and profiles are shown in figures 7(A) and (B) for the wide-FOV and HR-HS configurations, respectively. The system offers an average sensitivity of 0.0341% over a wide FOV of 28 cm in diameter and 20 cm in length, and 0.0626% over a focused FOV of 16 cm in diameter and 14 cm in length. It is worth mentioning that since CZT presents a linear attenuation of 0.354 mm^−1^ at 140 keV, the 1 cm crystal grants a probability of interaction of ∼97% at 140 keV that naturally assures a superior system sensitivity.

**Figure 8.**
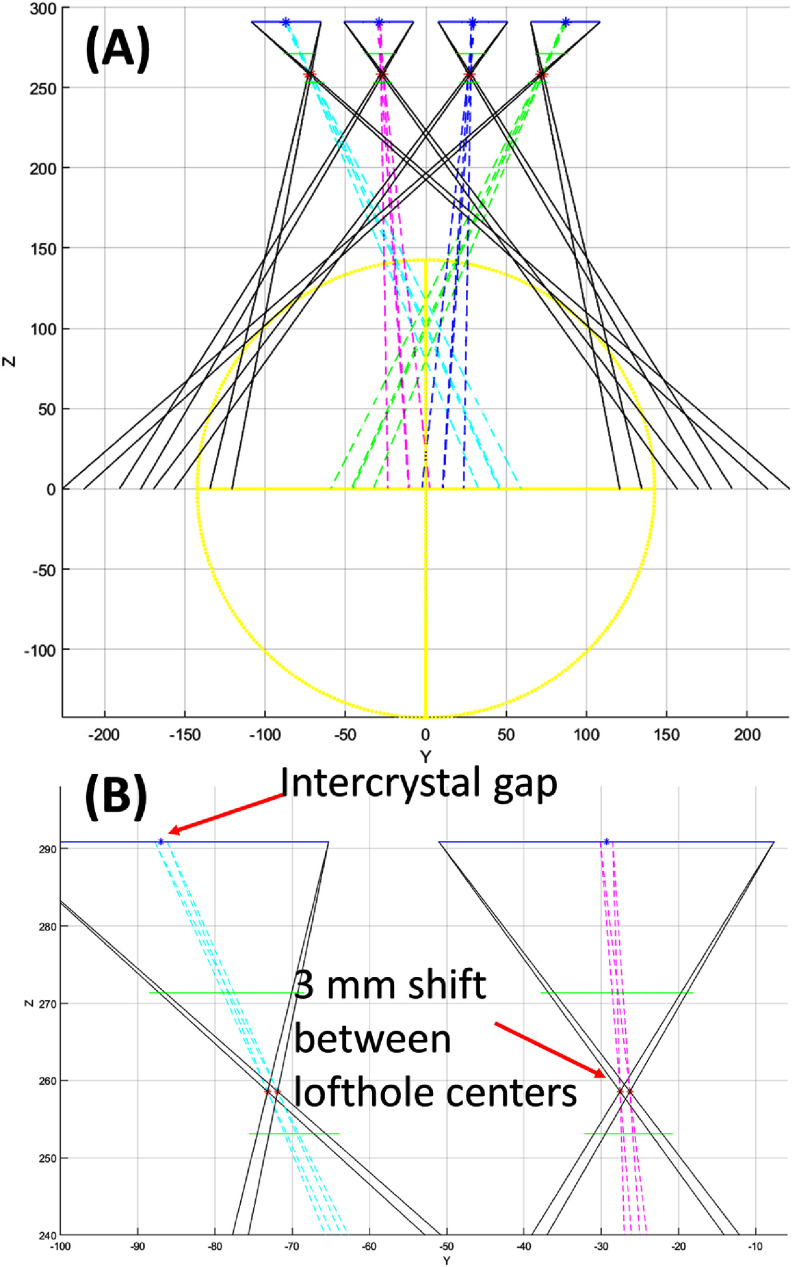
(A) Details of the wide-FOV collimator design showing the 3 mm shift of the lofthole centers. The colored dashed lines show where the intercrystal gaps in the detector units backproject in the object space. (B) Zoomed view of the 3 mm shift between lofthole centers.

As expected, the missing detectors in the bottom opening affect the uniformity of the system sensitivity in both configurations. In figure 7(A), the wide-FOV configuration shows valleys in sensitivity due to the presence of physical gaps between the detector crystals, since a single lofthole projects on 2 × 2 crystals inside a detector module (figure 4(C)). To reduce such non-uniformities, a shift of 3 mm has been introduced between the lofthole centers of adjacent rings along the axial direction, as shown in figure 8. The colored dashed lines in figure 8 show where the inter-crystal gaps in the detector units backproject in the object space. Due to the 3 mm lofthole shift (zoomed view in figure 8(B)), the under-sampled areas are spread out over a central region of 11 cm, divided into 8 subsections. Each subsection is seen by 7 loftholes out of 8 available in each detector panel, and 42 out of 48 in the entire system. This design assures that areas not sampled by several loftholes do not overlap in the object space and, therefore, this does not reduce considerably the local sensitivity. With this design, the depth of the deepest sensitivity valley results to be 0.004% (figure 7(A), last row), equal to a ∼11.7% variation in the average sensitivity of 0.0341%, over the entire wide FOV.

### 3.3. Imaging performance evaluation

**Figure 9.**
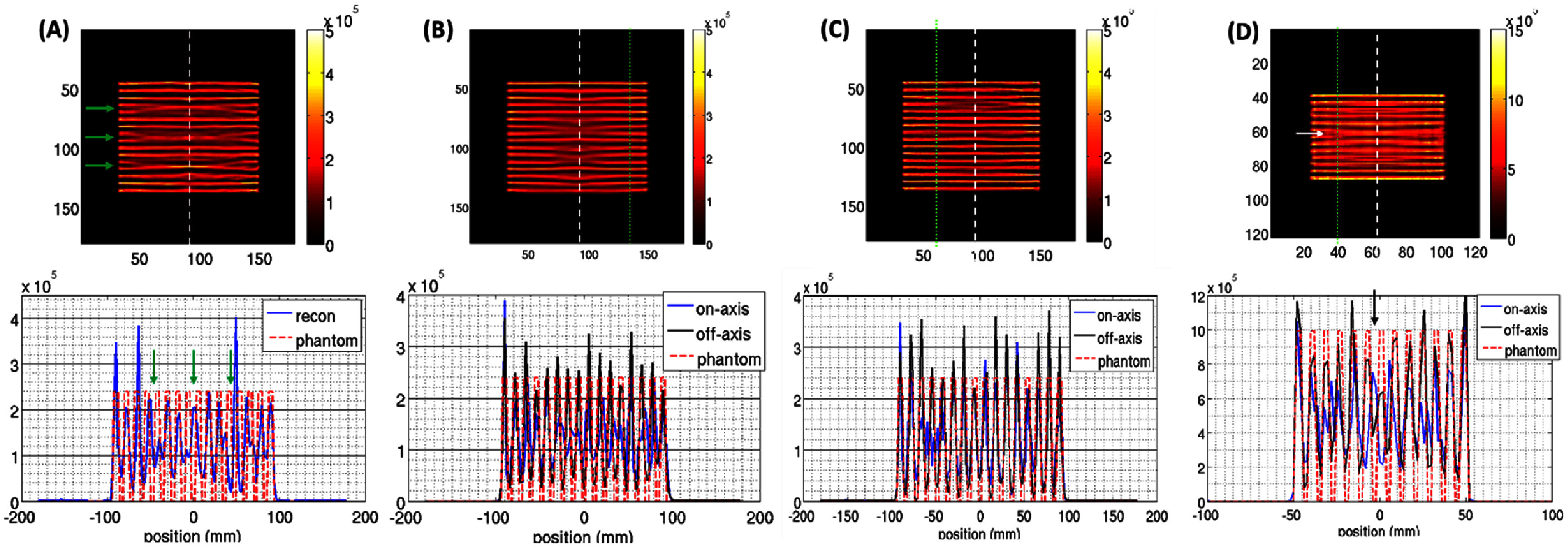
Defrise phantom reconstruction for axial sampling (noiseless conditions): (A) single image configuration in the wide-FOV configuration (phantom with 6 mm disks and spacings); (B) single image configuration in the wide-FOV configuration with two axial positions (phantom at the axial center, and 2 cm axial translation); (C) joint image reconstruction; (D) single image configuration in the HR-HS-FOV configuration (phantom with 4 mm thick disks and spacings). From the 180 × 180 voxels in the slice, we select the central 120 × 120 to enhance the visualization. The on-axis profile (blue line) is shown by the dashed white line, the off-axis (black line) by the green dotted line.

The noiseless image reconstructions of the Defrise phantom in wide-FOV mode are shown in figure 9(A-C) under three different reconstruction conditions: (1) *single mode reconstruction* (figure 9(A)): the reconstructed phantom (top), and central axial profile (bottom) shows that the wide-FOV configuration can effectively resolve most of 6 mm thick disks, but some deformations are introduced (green arrows) in areas affected by axial under-sampling (Zeraatkar *et al* 2020); (2) *2 cm axial translation* (figure 9(B)): to increase the number of acquired axial samples, we repeated the single mode reconstruction using two axial positions, one with the Defrise phantom at the axial center (same as (1)), and the other with 20 mm axial translation (∼dimension of a single CZT crystal). In this case, the axial sampling is improved showing all 16 disks at the periphery of the cylinder, but a few deformations are still visible along the phantom axis. (3) *joint reconstruction* (figure 9(C)): the phantom is reconstructed using the joint image method described in (5) and all the 16 6 mm disks are visible.

For the HR-HS configuration (figure 9(D)), all 4 mm thick disks are visible except for an under-sampled area at the center of the FOV (white arrow) that could be recovered with an axial translation of the object (Chen *et al* 2018, 2020, Zeraatkar *et al* 2020).

**Figure 10.**
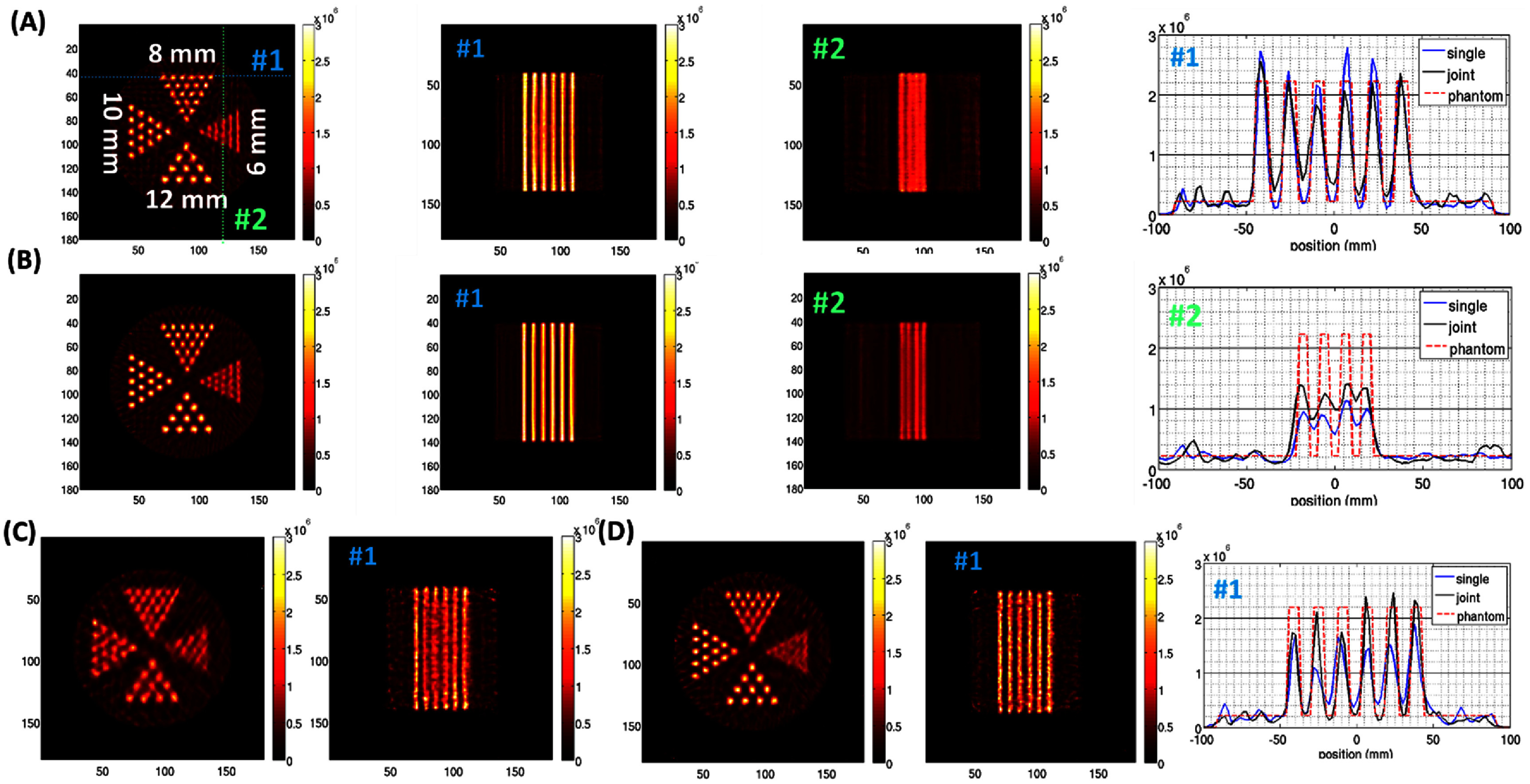
Digital hot-rod phantom in wide-FOV configuration: image reconstruction under (A)–(B) noiseless and (C)–(D) noisy conditions, showing (left) transaxial and (central and right) axial views. The blue and green dotted lines in the transaxial plane show the locations of axial views #1 and #2, respectively. These sections are the same for all the reconstructions shown. The digital resolution phantom has a total 10 mCi Tc-99 m, Signal: Background 10:1, and it is imaged for 60 min in case of single mode reconstruction (A)–(C), 30 min in wide-FOV and 30 min in HR-HS in case of joint reconstruction (B)–(D). No filter or regularization are applied. Same colorbar is used to plot all the images.

**Figure 11.**
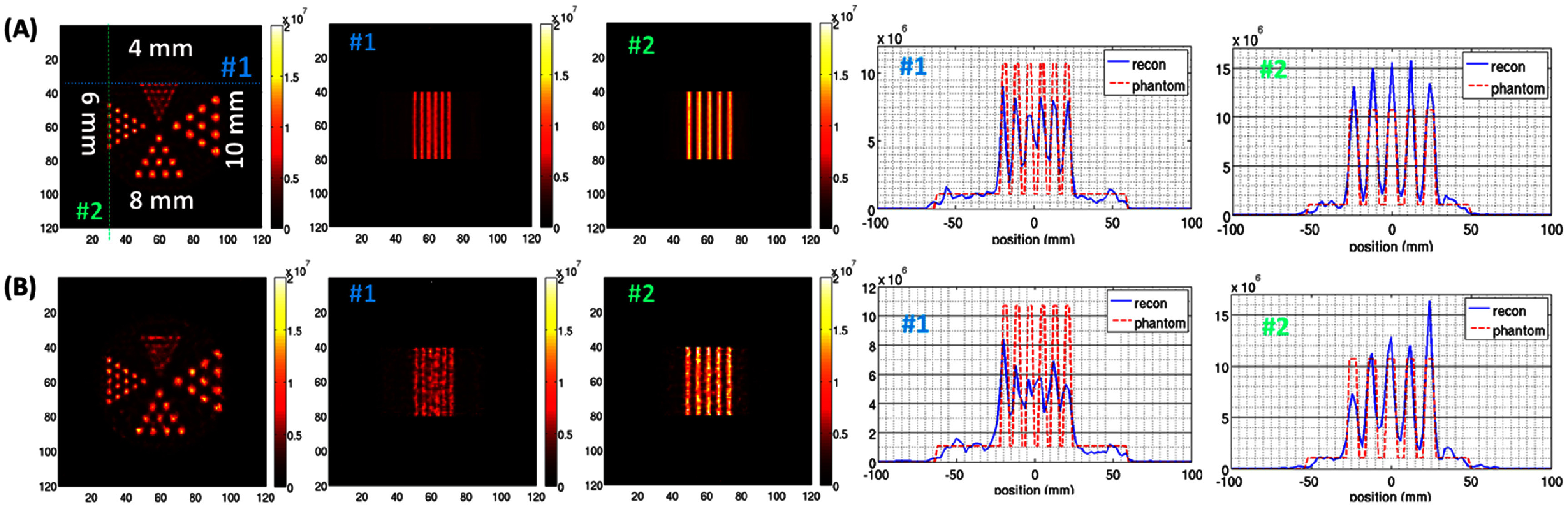
Digital hot-rod phantom in HR-HS configuration: image reconstruction under (A) noiseless and (B) noisy conditions, showing (1st column) transaxial and (2nd and 3rd columns) axial views (sections #1 and #2 are shown by the blue and green dotted lines, respectively). (4th and 5th columns) Line profiles for the 4 mm and 6 mm D hot rods. The digital resolution phantom has a total 2.5 mCi Tc99m, Signal:Background 10:1, and it is imaged for 60 min. No filter or regularization are applied. Same colorbar is used to plot all the images. The phantom consists of 180 × 180 × 180 voxels but to enhance the visualization, the central 120 × 120 area in the slice is shown.

Figures 10 and 11 show the image reconstruction of the hot-rod phantoms described in 2.4.3B under noiseless (figures 10(A), (B) and 11(A)) and noisy (figures 10(C), (D) and 11(B)) conditions. Figures 10(B)–(D) show the reconstruction of the resolution phantom when the joint reconstruction in (5) is applied. In this case the image acquisition is simulated for 30 min in the wide-FOV configuration and 30 min in the HR-HS configuration. From visual inspection, in wide-FOV configuration the smallest hot-rods that can be resolved are the 6 mm diameter under noiseless conditions (figure 10(A)), and 8 mm diameter under noisy conditions (figure 10(C)). Based on the design shown in figure 8 and described at the end of 3.2, these results demonstrate that even if the central area is not sampled by all 48 loftholes available in the system due to the intercrystal gaps in the wide-FOV configuration, the angular sampling is adequate and allows to reconstruct the features in the hot rod phantom. An evident improvement in image quality can be appreciated in both conditions when joint reconstruction is applied (figures 10(B) and (D)). An improvement, in contrast, is also visible in the profiles from the most external line of 8 mm hot rods (section#1 in figures 10(B) and (D)), and for the 4th line from the center of the 6 mm hot rods (section#2 in figure 10(B)). Similarly, in the HR-HS configuration the 4 mm diameter hot-rod are distinguishable if noiseless (figure 11(A)), 6 mm diameter if noisy (figure 11(B)).

**Figure 12.**
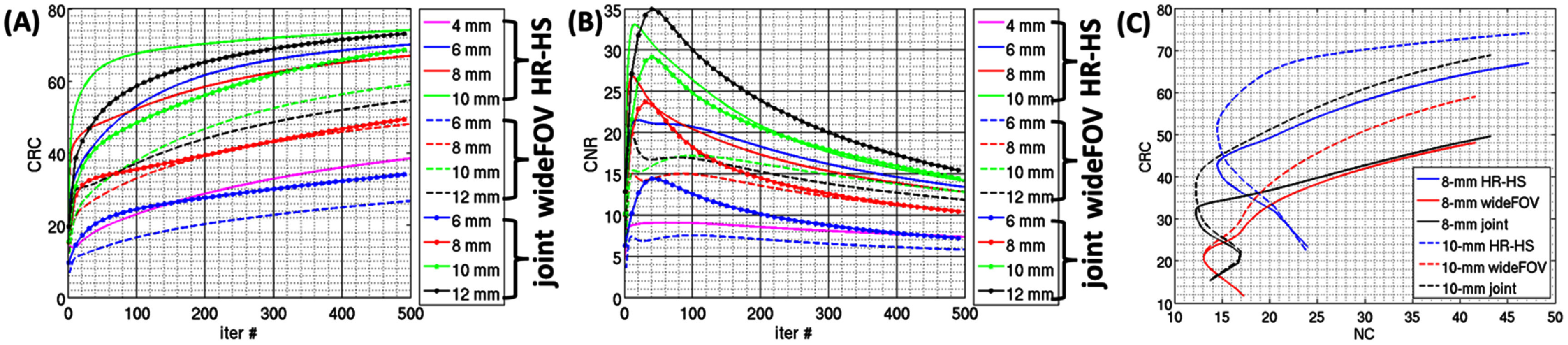
Capability of the DE-SPECT system to reproduce the contrast in the object: (A) CRC and (B) CNR versus iteration number for each hot-rod diameter in both imaging configurations (HR-HS solid lines, wide-FOV dashed lines, joint reconstruction dotted lines). The hot-rods are the ones closest to the axial center of the phantom in figure 10(C)/(D)–11(B). (C) Comparison of the CRC-NC curves for the 8 mm and 10 mm diameter hot-rods in the HR-HS mode (blue), wide-FOV mode (red) and joint reconstruction (black).

To assess the capability of the DE-SPECT system to reproduce the contrast in the object, we computed the CNR and CRC values, defined in (7) and (8), across 500 noisy iterations for the hot-rod of each group (the one located closest to the phantom axis) in both imaging configurations (figures 10(C) and 11(B)) and joint reconstruction (figure 10(D)). As visible in the comparative plots in figures 12(A) and (B), the HR-HS mode (solid lines) achieves higher CNR and CRC values for all the hot-rod diameters in comparison to the wide-FOV mode (dashed lines), even if no hot-rod achieves the complete contrast recovery (i.e. CRC of 100%). On the other side, it is worth noting that even if the HR-HS configuration reaches the highest contrast recovery, this is achieved with a higher noise amplification than the wide-FOV configuration, as visible in the CRC-NC curves estimated on the 8 mm and 10 mm hot-rods (figure 12(C)). Secondly, the CNR and CRC values from the joint reconstruction (dotted lines) are comparable to the values from the wide-FOV reconstruction (dashed lines) for the 8 mm hot-rods, while the first consistently outperform the latter for larger hot-rods diameters. These results showed that while the wider FOV coverage will reduce truncation artifacts for structures outside the ROI, the joint reconstruction will improve its quantitative performance.

## 4. Discussion

The DE-SPECT system introduces two important innovations in a SPECT scanner for imaging PVD in lower extremities:

(a) the use of *1 cm thick CZT spectrometers with DOI response* and sub-0.75 mm intrinsic resolution: the crystal thickness of 1 cm greatly improves the material efficiency (∼97% probability of interaction at 140 keV), and therefore, the system sensitivity, whereas the sub-0.75 mm intrinsic spatial resolution along *X-, Y*- directions assures a high reconstructed imaging resolution. The excellent spectroscopic performance over a broad energy spectrum (<2 keV at 140, 3.3 keV at 440 keV, figure 6) makes the DE-SPECT system the first full spectral imaging system for broadband imaging applications (50–600 keV). Additionally, the modularity of the detection units allows to explore non-conventional geometries where the detectors are arranged is a non-continous surface, such as the proposed checkerboard pattern. This allows to expand the total volume covered in the object space while providing adequate angular sampling and imaging performance.
(b) *The dynamic dual-FOV SCE collimator* is equipped with a computer-controlled mechanism that allows the user to choose between the two imaging configurations at any time during an imaging study. The compact design introduces a degree of flexibility without dramatically increasing the overall weight and footprint of the collimator and requires a relatively small axial translation of only 57 mm. The user will initially obtain preliminary information on the radiotracers’ distributions in a broad-band energy range in the 28 cm D × 20 cm L FOV (wide-FOV mode). Then the user will be able to focus on the ROI and to acquire detailed images with an improved sensitivity (peak central sensitivity 0.07% at 140 keV, figure 7(B)) and a ∼5 mm spatial resolution (figure 11(B)) inside the 16 cm D × 14 cm L FOV. This will allow to design optimized imaging acquisition protocols, and to reduce the overall imaging time, required dose and patient discomfort. Additionally, this allows to perform joint image reconstructions improving the imaging performance in the wide-FOV configuration while reducing truncation artifacts and data insufficiency due to the limited angular and axial sampling of the stationary geometry. While the wide-FOV configuration will be used to obtain a scout image, the HR-HS configuration presents quantitative improvements in the fidelity of the image reconstruction, in terms of higher CRC and CNR (figure 12), in agreement with the visual enhancement and better spatial resolution (figure 11). Under similar minification conditions (1:8 in both collimator designs), the apparent improvements between the two imaging configurations can be justified with the increased angular and axial sampling (192 loftholes in HR-HS VS 48 in wide-FOV) and with the increased sensitivity in the smaller FOV. As a comparison, the GE Discovery NM530c, a well-established stationary CZT-based cardiac stationary SPECT scanner (5.4% FWHM at 140 keV (Bocher *et al* 2010)), presents a peak sensitivity of 0.046%, 6.7 mm central spatial resolution and a spherical FOV of 19 cm in diameter (Imbert *et al* 2012). With ∼30% less CZT active area (1216 cm^2^ in NM530c VS 839 cm^2^ in DE-SPECT), the DE-SPECT offers an improved angular sampling (48/192 independent cameras VS 19 pinholes in NM530c) and superior imaging flexibility in two stationary configurations.

It is worth mentioning that the system can be easily expanded to have all 96 detector slots populated, for a total detection area of 1678 cm^2^. In this case, both the collimator apertures can be used simultaneously since the collimator design does not allow for multiplexing.

The reconstructions in the axial (figure 9) and trans-axial (figures 10 and 11) plane show that both imaging configurations presents insufficient angular and axial sampling, introducing evident distortions in the reconstructions (especially in the Defrise phantom in figure 9). Data sufficiency is a fundamental acquisition aspect in SPECT imaging that has been extensively studied (Smith 1985, Ter-Antonyan *et al* 2007, Clackdoyle and Noo 2020). Under-sampling is an inevitable condition in static configurations like the one adopted in the DE-SPECT system, where no translation or rotation around the object is possible. According to the historical paper (Rosenthal *et al* 1995), for 360° SPECT the optimal number of acquired projections should be:

\begin{align*}N = {\raise0.7ex\hbox{${\pi D}$} \!\mathord{\left/ {\vphantom {{\pi D} {{\text{d}}x}}}\right.} \!\lower0.7ex\hbox{${{\text{d}}x}$}}\end{align*}

where $N$ is the number of projection angles, $D$ is the diameter of the FOV and ${\text{d}}x$ is the smallest linear dimension that can be resolved. In the wide-FOV configuration *N*= 146 (28 cm diameter and 6 mm resolution) and *N* = 125 (16 cm diameter and 4 mm resolution) for the HR-HS. This would require 146 and 125 pinholes, respectively, equally spaced over 360° for each ring of the system, which is not currently feasible. Similarly, other commercially available static SPECT systems do not satisfy this condition, such as the GE Discovery NM 530c (Bocher *et al* 2010) that presents a 180° angular coverage of the heart in a 19 cm diameter spherical FOV with 19 pinholes. Nonetheless, the proposed DE-SPECT geometry can reconstruct features down to 4 mm (HR-HS) and 6 mm (wide-FOV), both in the axial (figure 9) and transaxial (figure 10) plane.

This is possible thanks to the proposed SCE collimator that overall improves the angular sampling of the static DE-SPECT design and alleviates the data insufficient condition. Additionally, the proposed joint reconstruction method further reduces the effects of the under-sampling as shown in figures 9(C) and 10(B)–(D), since it combines the information from the 24 angular views in the wide-FOV with the 48 angular views in the HR-HS.

In future work, we will experimentally explore the trans-axial and axial under-sampling in the DE-SPECT system. We will rotate and translate imaging phantoms to determine the optimal scanning procedure, and we will compare the reconstructions with the static case. We will also consider translating the collimator panels in intermediate axial positions (less than 57 mm), in order to increase the axial sampling of the system. This can be achieved using the position-sensitive actuators in the collimator, without interfering with the patient in the imaging state.

## 5. Conclusions

The DE-SPECT system is a high-performance SPECT system that offers a unique non-invasive diagnostic approach for the comprehensive assessment of molecular and pathophysiological changes in the lower extremities of patients with suspected PVD. In this work, we reported the research efforts in the design and development of the DE-SPECT system and evaluated the expected imaging performance through simulations.

The DE-SPECT system is the first full clinical spectral SPECT imaging system based on 3D position-sensitive 1 cm thick CZT detectors for lower extremities. The system offers an excellent spectroscopic performance (2.6 keV FWHM at 218 keV) that makes it an ideal platform to perform multi-tracer SPECT studies for PVD applications. Due to the complex and multifactorial nature of PVD, the DE-SPECT system will allow the exploration of novel molecular imaging strategies and the investigation of the interplay of concomitant pathophysiological processes radiolabeled with different tracers (Stacy 2022). The DE-SPECT imaging geometry was designed for optimized on-the-fly acquisition protocols during PVD studies (28 cm diameter FOV for dual-leg imaging, 16 cm diameter FOV for single-leg), offering a relatively high sensitivity and spatial resolution (0.07% peak and ∼5 mm in HR-HS mode). While the bottom opening of the gantry is not ideal due to the introduction of non-uniformities in sensitivity, it is required for the use of an inflatable mattress to effortlessly insert and position the patient’s leg(s). Finally, a consistent design effort was spent to ensure easy maneuverability and integration. The stationary system is installed on a portable wheeled superstructure, and it is meant to be integrated with a pre-existing cardiac SPECT(/CT) scanner to perform simultaneous imaging of the lower extremities and the heart. Rest and stress dynamic cardiac imaging can be used to provide image-derived input functions that, when combined with dynamic lower extremity imaging, enable radiotracer kinetic modeling and determination of absolute tissue flow and flow reserve in the lower extremities.

The DE-SPECT system is currently under fine-tuning and characterization. A detailed geometric calibration is needed to experimentally model the SRF (Lai *et al* 2020), to capture potential hardware misalignments, detector performance non-uniformities and to correctly estimate the system sensitivity. Future works will focus on the experimental validation of the simulation results. This will require careful consideration and implementation of several corrections, typically needed in SPECT imaging.

In terms of attenuation correction, we will consider the possibility of performing a CT scan of the extremities with the pre-existing SPECT/CT scanner used for cardiac imaging and using the anatomical information to incorporate the attenuation correction.

In terms of scatter correction, we expect that the superior intrinsic properties of the detection unit will significantly reduce the amount of scattering in the projections, allowing us to select a narrow energy window around the photopeaks. We will consider implementing both traditional, such as the dual and triple energy window methods (Hutton *et al* 2011), and ML-based scatter correction methods.

The HR-HS configuration will suffer from truncation artifacts due to the smaller FOV covered. Truncation artifacts are typically found in organ-specific imaging systems, such as in cardiac SPECT (Mao and Zeng 2013). We will implement correction methods in the imaging reconstruction to alleviate these effects. We believe that the proposed joint reconstruction method that incorporates information acquired in the wide-FOV configuration will be beneficial in reducing these artifacts.

Finally, other effects typical of CZT crystals need to be carefully considered and corrected. One of the major degrading effects is known as charge-sharing (Kim *et al* 2011, Veale *et al* 2014). We are extensively studying this phenomenon and we previously proposed a ML-based correction method (Yang *et al* 2023). We will further demonstrate the excellent spectroscopic performance of the CZT spectrometers in multi-functional and multi-tracer SPECT studies and explore the possibility to perform spatial-spectral joint image reconstructions.

## Data Availability

The data cannot be made publicly available upon publication because no suitable repository exists for hosting data in this field of study. The data that support the findings of this study are available upon reasonable request from the authors.

## References

[pmbad5266bib1] Bocher M, Blevis I M, Tsukerman L, Shrem Y, Kovalski G, Volokh L (2010). A fast cardiac gamma camera with dynamic SPECT capabilities: design, system validation and future potential. Eur. J. Nucl. Med. Mol. Imaging.

[pmbad5266bib2] Carrino J A (2014). Dedicated cone-beam CT system for extremity imaging. Radiology.

[pmbad5266bib3] Chen Y, Goorden M C, Vastenhouw B, Beekman F J (2020). Optimized sampling for high resolution multi-pinhole brain SPECT with stationary detectors. Phys. Med. Biol..

[pmbad5266bib4] Chen Y, Vastenhouw B, Wu C, Goorden M C, Beekman F J (2018). Optimized image acquisition for dopamine transporter imaging with ultra-high resolution clinical pinhole SPECT. Phys. Med. Biol..

[pmbad5266bib5] Chou T H, Stacy M R (2020). Clinical applications for radiotracer imaging of lower extremity peripheral arterial disease and critical limb ischemia. Mol. Imaging Biol..

[pmbad5266bib6] Chun S Y, Nguyen M P, Phan T Q, Kim H, Fessler J A, Dewaraja Y K (2020). Algorithms and analyses for joint spectral image reconstruction in Y-90 bremsstrahlung SPECT. IEEE Trans. Med. Imaging.

[pmbad5266bib7] Chung M (2011). Emerging MRI technologies for imaging musculoskeletal disorders under loading stress. Appendix F, Dedicated Extremity MRI Devices.

[pmbad5266bib8] Clackdoyle R, Noo F (2020). Quantification of tomographic incompleteness in cone-beam reconstruction. IEEE Trans. Radiat. Plasma Med. Sci..

[pmbad5266bib9] Deprez K, Pato L R V, Vandenberghe S, Van Holen R (2013). Characterization of a SPECT pinhole collimator for optimal detector usage (the lofthole). Phys. Med. Biol..

[pmbad5266bib10] Efthimiou N, Karp J S, Surti S (2022). Data-driven, energy-based method for estimation of scattered events in positron emission tomography. Phys. Med. Biol..

[pmbad5266bib11] Frey E C, Tsui B M W, Zaidi H (2006). Collimator-detector response compensation in SPECT. Quantitative Analysis in Nuclear Medicine Imaging.

[pmbad5266bib12] González A J, Sánchez F, Benlloch J M (2018). Organ-dedicated molecular imaging systems. IEEE Trans. Radiat. Plasma Med. Sci..

[pmbad5266bib13] He Z, Li W, Knoll G F, Wehe D K, Berry J, Stahle C M (1999). 3-D position sensitive CdZnTe gamma-ray spectrometers. Nucl. Instrum. Methods Phys. Res. A.

[pmbad5266bib14] Hudson H M, Larkin R S (1994). Accelerated image reconstruction using ordered subsets of projection data. IEEE Trans. Med. Imaging.

[pmbad5266bib15] Hutton B F, Buvat I, Beekman F J (2011). Review and current status of SPECT scatter correction. Phys. Med. Biol..

[pmbad5266bib16] Imbert L, Poussier S, Franken P R, Songy B, Verger A, Morel O, Wolf D, Noel A, Karcher G, Marie P-Y (2012). Compared performance of high-sensitivity cameras dedicated to myocardial perfusion SPECT: a comprehensive analysis of phantom and human images. J. Nucl. Med..

[pmbad5266bib17] Karimeddini D, Bergmann S (2016). The state of the future is solid. J. Nucl. Cardiol..

[pmbad5266bib18] Kim J C, Anderson S E, Kaye W, Zhang F, Zhu Y, Kaye S J, He Z (2011). Charge sharing in common-grid pixelated CdZnTe detectors. Nucl. Instrum. Methods Phys. Res. A.

[pmbad5266bib19] Kruijff R M D, Raavé R, Kip A, Molkenboer-Kuenen J, Morgenstern A, Bruchertseifer F, Heskamp S, Denkova A G (2019). The in vivo fate of 225Ac daughter nuclides using polymersomes as a model carrier. Sci. Rep..

[pmbad5266bib20] Lai X, Meng L-J (2018). Simulation study of the second-generation MR-compatible SPECT system based on the inverted compound-eye gamma camera design. Phys. Med. Biol..

[pmbad5266bib21] Lai X, Petibon Y, El Fakhri G, Ouyang J (2018). Joint reconstruction of rest/stress myocardial perfusion SPECT. Phys. Med. Biol..

[pmbad5266bib22] Lai X, Zannoni E M, George J, Meng L-J (2020). System modeling and evaluation of a prototype inverted-compound eye gamma camera for the second generation MR compatible SPECT. Nucl. Instrum. Methods Phys. Res. A.

[pmbad5266bib23] Mao Y, Zeng G L (2013). A tailored ML-EM algorithm for reconstruction of truncated projection data using few view angles. Phys. Med. Biol..

[pmbad5266bib24] Meng L-J, Lai X, Zannoni E M (2022). Gamma camera for spect imaging and associated methods. U.S. Patent.

[pmbad5266bib25] Nelson B J B, Andersson J D, Wuest F (2020). Targeted alpha therapy: progress in radionuclide production, radiochemistry, and applications. Pharmaceutics.

[pmbad5266bib26] Ocak M, Toklu T, Demirci E, Selçuk N, Kabasakal L (2020). Post-therapy imaging of 225Ac-DOTATATE treatment in a patient with recurrent neuroendocrine tumor. Eur. J. Nucl. Med. Mol. Imaging.

[pmbad5266bib27] Pollak A W, Norton P T, Kramer C M (2012). Multimodality imaging of lower extremity peripheral arterial disease: current role and future directions. Circ.: Cardiovascular Imaging.

[pmbad5266bib28] Popescu L M, Lewitt R M, Matej S, Karp J S (2006). PET energy-based scatter estimation and image reconstruction with energy-dependent corrections. Phys. Med. Biol..

[pmbad5266bib29] Press W H, Teukolsky S A, Vetterling W T, Flannery B P (2007). Numerical recipes. The Art of Scientific Computing.

[pmbad5266bib30] Rakvongthai Y, Fahey F, Borvorntanajanya K, Tepmongkol S, Vutrapongwatana U, Zukotynski K, El Fakhri G, Ouyang J (2017). Joint reconstruction of ictal/inter-ictal SPECT data for improved epileptic foci localization. Med. Phys..

[pmbad5266bib31] Rosenthal M S, Cullom J, Hawkins W, Moore S C, Tsui B M W, Yester M (1995). Quantitative SPECT imaging: a review and recommendations by the focus committee of the society of nuclear medicine computer and instrumentation council. J. Nucl. Med..

[pmbad5266bib32] Roser J (2022). Joint image reconstruction algorithm in compton cameras. Phys. Med. Biol..

[pmbad5266bib33] Sankar P, Zannoni E M, Liu C, Sinusas A, Meng L J, Metzler S (2022). Evaluation of DE-SPECT pinholes exchange adaptive mechanism. J. Nucl. Med..

[pmbad5266bib34] Scheinberg D A, McDevitt M R (2011). Actinium-225 in targeted alpha-particle therapeutic applications. Curr. Radiopharm..

[pmbad5266bib35] Seo Y (2019). Quantitative imaging of alpha-emitting therapeutic radiopharmaceuticals. Nucl. Med. Mol. Imaging.

[pmbad5266bib36] Shabani Varaki E, Gargiulo G D, Penkala S, Breen P P (2018). Peripheral vascular disease assessment in the lower limb: a review of current and emerging non-invasive diagnostic methods. Biomed. Eng. Online.

[pmbad5266bib37] Sharir T, Slomka P J, Berman D S (2010). Solid-state SPECT technology: fast and furious. J. Nucl. Cardiol..

[pmbad5266bib38] Shu J, Santulli G (2018). Update on peripheral artery disease: epidemiology and evidence-based facts. Atherosclerosis.

[pmbad5266bib39] Smith B D (1985). Image reconstruction from cone-beam projections: necessary and sufficient conditions and reconstruction methods. IEEE Trans. Med. Imaging.

[pmbad5266bib40] Stacy M R (2022). Molecular imaging of lower extremity peripheral arterial disease: an emerging field in nuclear medicine. Front. Med. Perspect..

[pmbad5266bib41] Stacy M R, Sinusas A J (2016). Novel applications of radionuclide imaging in peripheral vascular disease. Cardiol. Clin..

[pmbad5266bib42] Stacy M R, Zhou W, Sinusas A J (2013). Radiotracer imaging of peripheral vascular disease. J. Nucl. Med..

[pmbad5266bib43] Sutter R, Tresch F, Buck F M, Pfirrmann C W A (2014). Is dedicated extremity 1.5-T MRI equivalent to standard large-bore 1.5-T MRI for foot and knee examinations?. Am. J. Roentgenol..

[pmbad5266bib44] Ter-Antonyan R, Jaszczak R J, Bowsher J E, Greer K L, Metzler S D (2007). Half-cone-beam data sufficiency in triple-camera SPECT.

[pmbad5266bib45] Van Audenhaege K, Vandenberghe S, Deprez K, Vandeghinste B, Van Holen R (2013). Design and simulation of a full-ring multi-lofthole collimator for brain SPECT. Phys. Med. Biol..

[pmbad5266bib46] Veale M C, Bell S J, Duarte D D, Schneider A, Seller P, Wilson M D, Iniewski K (2014). Measurements of charge sharing in small pixel CdTe detectors. Nucl. Instrum. Methods Phys. Res. A.

[pmbad5266bib47] Yang C, Zannoni E M, Meng L-J (2023). Joint estimation of interaction position and energy deposition in semiconductor SPECT imaging sensors using fully connected neural network. Phys. Med. Biol..

[pmbad5266bib48] Zannoni E M (2022). The DE-SPECT System: the first clinical SPECT system for broadband multi-isotope imaging of the lower extremities. J. Nucl. Med..

[pmbad5266bib49] Zannoni E M (2023). In vivo 3-D gamma-ray spectrometry for multifunctional molecular imaging and theragnostic in lower extremities. J. Nucl. Med..

[pmbad5266bib50] Zannoni E M, Yang C, Meng L J (2021). Design study of an ultrahigh resolution brain SPECT system using a synthetic compound-eye camera design with micro-slit and micro-ring apertures. IEEE Trans. Med. Imaging.

[pmbad5266bib51] Zeraatkar N, Kalluri K S, Auer B, Konik A, Fromme T J, Furenlid L R, Kuo P H, King M A (2020). Investigation of axial and angular sampling in multi-detector pinhole-SPECT brain imaging. IEEE Trans. Med. Imaging.

[pmbad5266bib52] Zhang F, Herman C, He Z, Geronimo G D, Vernon E, Fried J (2012). Characterization of the H3D ASIC readout system and 6.0 cm^3^ 3-D position sensitive CdZnTe detectors. IEEE Trans. Nucl. Sci..

[pmbad5266bib53] Zhang F, Kaye W R, He Z (2009). Performance of 3-D position sensitive CdZnTe detectors for gamma-ray energies above 1 MeV.

[pmbad5266bib54] Zhu Y, Anderson S E, He Z (2011). Sub-pixel position sensing for pixelated, 3-D position sensitive, wide band-gap, semiconductor, gamma-ray detectors. IEEE Trans. Nucl. Sci..

